# Retraction of WHO guidance on opioid use

**DOI:** 10.2471/BLT.19.249417

**Published:** 2020-01-01

Further to concerns raised in 2019, WHO Director-General Dr Tedros ordered a review of two guidance documents on pain control. The resulting statement, which appears below in full, was published on 20 June 2019 on WHO’s website (https://www.who.int/medicines/areas/quality_safety/guide_on_pain/en/). 

“WHO takes very seriously concerns recently raised about the development of its 2011 guidance *Ensuring balance in national policies on controlled substances: guidance for availability and accessibility of controlled medicines*^1^, as well as its 2012 *WHO guidelines on the pharmacological treatment of persisting pain in children with medical illnesses*.^2^

WHO is discontinuing these guidelines in light of new scientific evidence that has emerged since the time of their publication. This will also address any issues of conflicts of interest of the experts that have been raised.

WHO remains fully committed to ensuring that people suffering severe pain have access to effective pain relief medication, including opioids. WHO is concerned that there is very low access to medication for moderate and severe pain, particularly in low and middle-income countries.

WHO also recognizes that the need for access to pain relief must be balanced with concerns about the harm arising from the misuse of medications prescribed for the management of pain, including opioids. Scientific evidence indicates there are risks associated with the use of these medications —such as the development of dependence, overdose and accidental death. Even when prescribed according to established clinical guidelines and patients’ needs, and used as directed, certain factors may increase these risks.

While potential harms can be reduced through enforcement of proper regulation of controlled medicines, careful initial assessment of patients prior to prescribing, and regular patient monitoring and patient education, the differences between acute and chronic pain need to be understood and managed accordingly. Recent research in the fields of palliative care and pain management has identified many strategies for managing pain, beyond drug treatment alone. Evaluating this new evidence and establishing best strategies for alleviating pain—both acute and chronic—is an important area of work for WHO.

In this regard, WHO has already initiated the process for reviewing and updating its guidelines and policy documents regarding pain management and, in January 2019, published the new *WHO guidelines for the pharmacological and radiotherapeutic management of cancer pain in adults and adolescents*^3^, available here: https://www.who.int/ncds/management/palliative-care/cancer-pain-guidelines/en/. Further work to produce guidance for management of pain in different age groups is ongoing, including the review of guidance for children. Information on the revision process can be found on WHO’s website at: https://www.who.int/news-room/detail/27-08-2019-who-revision-of-pain-management-guidelines.

WHO remains committed to working with Member States to support the development of evidence-based policies, regulations and best practices to promote access to safe, effective and affordable medicines for the management of pain, and to prevent their misuse and harm.”
